# Mild traumatic brain injury is associated with dysregulated neural network functioning in children and adolescents

**DOI:** 10.1093/braincomms/fcab044

**Published:** 2021-03-17

**Authors:** Kristina Safar, Jing Zhang, Zahra Emami, Avideh Gharehgazlou, George Ibrahim, Benjamin T Dunkley

**Affiliations:** 1 Department of Diagnostic Imaging, Hospital for Sick Children, Toronto, ON, Canada M5G 0A4; 2 Neurosciences & Mental Health, SickKids Research Institute, Toronto, ON, Canada M5G 0A4; 3 Institute of Medical Science, Faculty of Medicine, University of Toronto, Toronto, ON, Canada M5S 1A8; 4 Department of Surgery, University of Toronto, Toronto, ON, Canada M5T 1P5; 5 Institute of Biomaterials and Biomedical Engineering, University of Toronto, Toronto, ON, M5S 3G9 Canada; 6 Department of Medical Imaging, University of Toronto, Toronto, ON, Canada M5T 1W7

**Keywords:** mild traumatic brain injury, functional connectivity, magnetoencephalography, machine learning classification, children

## Abstract

Mild traumatic brain injury is highly prevalent in paediatric populations, and can result in chronic physical, cognitive and emotional impairment, known as persistent post-concussive symptoms. Magnetoencephalography has been used to investigate neurophysiological dysregulation in mild traumatic brain injury in adults; however, whether neural dysrhythmia persists in chronic mild traumatic brain injury in children and adolescents is largely unknown. We predicted that children and adolescents would show similar dysfunction as adults, including pathological slow-wave oscillations and maladaptive, frequency-specific, alterations to neural connectivity. Using magnetoencephalography, we investigated regional oscillatory power and distributed brain-wide networks in a cross-sectional sample of children and adolescents in the chronic stages of mild traumatic brain injury. Additionally, we used a machine learning pipeline to identify the most relevant magnetoencephalography features for classifying mild traumatic brain injury and to test the relative classification performance of regional power versus functional coupling. Results revealed that the majority of participants with chronic mild traumatic brain injury reported persistent post-concussive symptoms. For neurophysiological imaging, we found increased regional power in the delta band in chronic mild traumatic brain injury, predominantly in bilateral occipital cortices and in the right inferior temporal gyrus. Those with chronic mild traumatic brain injury also showed dysregulated neuronal coupling, including decreased connectivity in the delta range, as well as hyper-connectivity in the theta, low gamma and high gamma bands, primarily involving frontal, temporal and occipital brain areas. Furthermore, our multivariate classification approach combined with functional connectivity data outperformed regional power in terms of between-group classification accuracy. For the first time, we establish that local and large-scale neural activity are altered in youth in the chronic phase of mild traumatic brain injury, with the majority presenting persistent post-concussive symptoms, and that dysregulated interregional neural communication is a reliable marker of lingering paediatric ‘mild’ traumatic brain injury.

## Teaser

Safar *et al*. show alterations in local and large-scale neural activity in children and adolescents with mild traumatic brain injury, with the majority experiencing long-term post-concussive symptoms, and establish that the functional connectome is a reliable and accurate classification marker for mild paediatric brain injury.

## Graphical Abstract

**Graphical Abstract:**
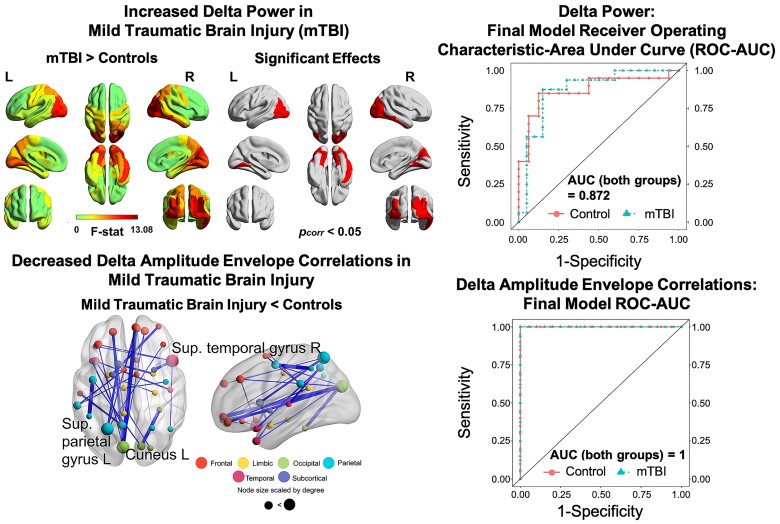

## Introduction

Mild traumatic brain injury (mTBI), commonly referred to as concussion, is a result of acceleration–deceleration forces following direct head contact or rotational forces of the head on the brain, such as whiplash.^1^^,^^2^ TBI-related visits to the emergency department are estimated to be as high as 692/100 000 in children 14 years and younger, accounting for just under a half million visits annually in the United States alone, with the bulk of cases classified as mild.^3^ Across the lifespan, mTBI is most prevalent in children and adolescents,^4^^,^^5^ with an estimated 11–29.3% of cases presenting persistent symptoms 3 months after injury.^6–9^ The biomechanical forces of mTBI are thought to impart diffuse axonal injury (DAI), which may involve cortical de-afferentation, disruption, axonal stretching and inflammation.^10^^,^^11^ Injury is often accompanied by acute symptoms (i.e. headache, dizziness, attention difficulties, fatigue, anxiety and irritability), which spontaneously resolve in around 70–80% of cases within 3 months.^12^ However, a substantial minority of injured individuals continue to experience chronic physical, cognitive and emotional impairments, known as persistent post-concussive symptoms (PPCS).^1^^,^^13–15^ Chronic PPCS in childhood and adolescence has been associated with reduced health-related quality of life,^16^^,^^17^ difficulties in school requiring intervention^17^ and may result in poorer long-term psychological and behavioural outcome.^18^

Neither mTBI nor PPCS have any clinical indicators on conventional structural imaging [i.e. magnetic resonance imaging (MRI), computed tomography (CT)]. Nevertheless, chronic symptoms suggest persistent neurophysiological dysfunction to which these techniques are blind. To understand the neuropathology of chronic mTBI non-invasively, neurophysiological techniques, including electroencephalography (EEG) and magnetoencephalography (MEG) are required. These techniques are a direct measure of neural activity, with a high temporal resolution, with MEG additionally affording an increased spatial resolution for precisely localizing neural functioning,^19^ including high-frequency neural oscillations. Neural oscillations and their dynamics play a crucial role in opening and closing temporal windows of opportunity for information processing in the brain,^20^ with oscillating neural ensembles operating at multiple spatial scales, from local microcircuits to large-scale brain-wide networks.^21^^,^^22^ Pathological slow-wave oscillations in the delta and theta frequency bands have been consistently observed in symptomatic adults with mTBI.^21^^,^^23–27^ The locus of abnormal slow-wave activity corresponds with de-afferenated and demyelinated cortical grey matter, as well as proximal axonal injury,^23^ and is correlated with neuropsychological measures in PPCS after mTBI.^27^

In addition to focal abnormalities, converging studies suggest that mTBI involves diffuse axonal injury, that involves widespread neurophysiological dysregulation, and network dysfunction and dysconnectivity.^21^^,^^28–32^ Alterations in functional connectivity in adults with mTBI have been reported in low-frequency (i.e. delta, theta, alpha) and gamma ranges, involving extended networks across multiple brain areas.^21^^,^^31^^,^^32^ For instance, using amplitude envelope coupling, Dunkley et al.^21^ reported widespread dysregulated functional networks in adults with injury in delta, theta and alpha frequency bands, primarily in the left hemisphere and encompassing frontal, temporal and subcortical brain areas. These network alterations were also directly correlated with cognitive and emotional symptoms in mTBI.

There have been few neurophysiological studies of chronic mTBI in children and adolescents.^33^ No studies have examined regional and distributed neural functioning in chronic mTBI in youth. The current MEG study investigated localized and large-scale neural dysrhythmia in a sample of youth with chronic mTBI against a group of typically-developing peers. We characterized focal neurophysiological disruption via regional power, and large-scale brain-wide networks using amplitude envelope correlations (AEC). The latter was selected as it is a reliable correlate of structural architecture^34^ and reflects the temporal coordination of intrinsic fluctuations in neural activity.^35^ Based on MEG findings in adults with mTBI, we hypothesized that children and adolescents with chronic mTBI would exhibit pathological slow-wave oscillations in the delta or theta range, as well as altered neural coupling, predominantly in frontal cortices, an area thought to be particularly susceptible to concussive injuries.^21^^,^^24–26^ Moreover, using an extension of our previous statistical learning-based classification tools,^36^ we aimed to apply these techniques in an analysis using a comprehensive multivariate pipeline to select the most relevant MEG features and to test the relative classification performance of regional power (capturing localized deficits due to focal injuries) versus functional coupling (indexing distributed and diffuse network injury) in children and adolescents with chronic mTBI. Based on our previous work showing that classification based on connectome data affords higher classification accuracy in individuals with mTBI than localized dysfunction,^36^ we predicted that dysregulation in the neurophysiological connectome would provide a richer feature set to reliably discriminate individual cases in children, too.

## Materials and methods

### Participants

Data were collected from 37 children and adolescents; 17 with chronic mTBI, with 1 excluded due to excessive head motion in the MEG, resulting in 16 participants included in the final analysis; and 20 age- and sex-matched typically developing peers as controls. The sample size was determined based on previous MEG studies of mTBI^21^^,^^32^^,^^33^ and those in the paediatric literature^37^^,^^38^ that have established significant effects with similarly sized samples. All participants were recruited between 2018 and 2020. Inclusion criteria for all participants included being between the ages of 6–18 years. For the chronic mTBI group, specific inclusion criteria included a diagnosis of mTBI determined by a physician and being in the chronic stage of injury. The mean time to scanning from day of injury was 455.56 days (∼15 months). Demographic characteristics are presented in Table 1.

**Table 1:** Demographic characteristics

	mTBI (*n *=* *16)	Control (*n *=* *20)	*P*-value^a^
Mean age in years (SD), range	12.46(3.24), 6.17–17.92	13.14(2.69), 6.31–15.74	0.369
Sex (*N* males)	11	10	0.257
Right-handedness (%)	81.3	87.5	
Mean time since injury to scan in days (SD), range	455.56(421.9), 79–1557	–	

a Comparisons by Mann–Whitney U-test for age and chi-square test for sex.

Symptomatology was assessed on the day of the scan using the Post-Concussion Symptom Inventory (PCSI; see Table 2). Exclusion criteria for all participants included permanent metal implants or implanted medical devices, a diagnosis of any other neurological disorder, intellectual disability, or taking medications that can directly modulate neural activity such as anticonvulsants, benzodiazepines, and/or GABA antagonists. All participants and their parents provided written informed assent/consent in accordance with the Declaration of Helsinki. The study was approved by the Research Ethics Board at the Hospital for Sick Children.

**Table 2:** Proportion of participants with PPCS

PPCS	Proportion of participants
Headache^a^	0.53
Fatigue^a^	0.53
Irritable	0.5
Difficulty concentrating	0.5
Feeling mentally ‘foggy’^a^	0.47
Dizziness	0.44
Drowsiness^a^	0.4
Sensitivity to light^a^	0.4
Sensitivity to noise^b^	0.36
Nausea^a^	0.34
Answer questions more slowly than usual^b^	0.29
Difficulty remembering^a^	0.27
Nervousness^a^	0.27
Vision problems (double vision, blurring)^a^	0.27
Sadness^a^	0.2
Balance problems^a^	0.2
Feeling slowed down^a^	0.13

a Missing data from one participant are not included.

b Missing data from two participants are not included.

### MEG data acquisition and pre-processing

Five-minute recordings of eyes-open resting-state MEG data were acquired using a 151 channel CTF system at a sampling rate of 600 Hz in a magnetically shielded room at the

Hospital for Sick Children, Toronto. Participants were lying in the supine position with the posterior of the head lying against the rear portion of the helmet, ensuring the greatest degree of head stability and minimized head motion. Fiducial coils situated at the left and right pre-auricular areas and nasion were used to continuously track head position throughout the run. The FieldTrip toolbox^39^ and in-house scripts written in MATLAB R2019a were used for MEG data pre-processing and source reconstruction.

The continuous data were filtered using a 4th order Butterworth band-pass filter at 1–150 Hz and a Fourier transform notch filter at the line utility of 60 and 120 Hz harmonic. Independent component analysis (ICA) was applied to the continuous signal to attenuate ocular and cardiac-related artefacts (‘fastica’ function in FieldTrip). Identified components were then removed manually following visual inspection of the MEG signal by an experienced analyst. The post-ICA artifact-free 5-minute recordings were epoched into 10 s segments. Additionally, epoch exclusion criteria included: Head motion more than 10 mm from the initial median head position during any given epoch, SQUID jumps in the MEG signal and/or signal jumps exceeding ±2000 fT. Based on the minimization and exclusion of artifacts, as many 10 s epochs as possible were retained for subsequent analyses.

There was no significant difference in the mean number of epochs included (after rejection) when contrasting groups, as determined by a Mann–Whitney U-test (PPCS: *M *= 18.5, SD = 5.73, Mdn = 20.5; controls: *M *= 20.1, SD = 0.45, Mdn = 20.5; *U *= 152, *z* = −0.27, *P *= 0.814). Head motion significantly differed between groups as determined by a Mann–Whitney U-test (PPCS: *M *= 1.83, SD = 1.79, Mdn = 1.21; controls: *M *= 0.64, SD = 0.51, Mdn = 0.54; *U *= 56.5, *z* = −3.29, *P *= 0.001); to account for this, head movement was used as a nuisance covariate in later MEG analyses.

For MEG data co-registration, a single-shell head model for each participant was generated based on an anatomical T_1_-weighted MRI template that was computed using SPM12 through FieldTrip (git commit: 93bf9d). A template MRI introduces no systematic bias or inconsistency into the source localization of the MEG signal when compared with a native MRI.^40^ Electrophysiological sources and connectivity can be robustly reconstructed using age-appropriate MRI templates in the absence of individual head models.^41^^,^^42^ The centroids of the first 90 parcels of the Automated Anatomical Labelling (AAL) atlas were used as seed/node locations, with the 90 AAL parcels comprising subcortical and cortical brain regions^43^ non-linearly warped onto equivalent locations in template space. The 90 regions of the AAL^43^ are widely-used as regions of interest (ROIs)/seed locations in many recent connectomic imaging studies,^44–47^ including neurophysiological studies of mTBI, both by our group^21^^,^^32^^,^^48^^,^^49^ and others.^29^^,^^50^ In addition, using 90-ROIs reduces dimensionality and computation time significantly, and the sparse seeding of the brain from using the 90 AAL centroids results in maximally independent virtual sensor time series as leakage (and therefore spurious ‘ghost’ interactions) are sufficiently attenuated by choosing such an atlas.^51^ A linearly constrained minimum variance (LCMV) beamformer^52^ was applied to reconstruct the broadband time series for the centroid of each of the AAL sources, with 5% Tikhonov regularization.

### Regional power spectral density

Following source reconstruction, the broadband time series data for each of the 90 AAL sources were *Z*-score normalized, and Welch’s method was used to compute regional power spectral density (PSD) (‘pwelch’ function in Matlab; MathWorks, Natick, USA). The PSD was calculated across each epoch and binned into canonical frequency bands: Delta (1–3 Hz), theta (4–7 Hz), alpha (8–14 Hz), beta (15–30 Hz), low gamma1 (30–55 Hz), low gamma2 (65–80 Hz) and high gamma (80–150 Hz). The average PSD across all epochs, in each frequency band, was then calculated for subsequent statistical analyses on power.

### Interregional AEC

The broadband time series data for each source was filtered into delta, theta, alpha, beta, low gamma1, low gamma2 and high gamma canonical frequency bands using a two-pass FIR filter (MATLAB’s fir1 and filtfilt). For functional connectivity, possible signal leakage between adjacent source-pairs was corrected for by using a symmetric orthogonalization procedure.^53^ The Hilbert transform was used (Hilbert envelope) to acquire the instantaneous amplitude envelope across each of the 10 s epochs, for each source and frequency band. Time series of the amplitude envelopes were subsequently temporally down-sampled to 1 Hz.^54^ Amplitude envelope correlations between each source-pair were acquired by calculating the Pearson correlation coefficient across an epoch, yielding a 90-by-90 connectivity matrix for each frequency band and participant, per epoch. These were subsequently averaged over the entire run to generate estimates of ‘static’ functional connectivity.

### Statistical analyses

To calculate group differences in regional PSD, a one-way ANCOVA (Factor Group, with two levels ‘mTBI’ and ‘controls’), with age and head motion as covariates, was computed for each of the 90 AAL sources, and the *P-*value was adjusted for multiple comparisons using the Benjamini–Hochberg correction for false discovery rate (FDR)-corrected, *P* < 0.05. To determine group differences in AEC, a one-way ANCOVA (Group: MTBI, controls), with age and head motion as covariates, was computed for individual AEC values in the 90-by-90 connectivity matrix to test for group differences in functional connectivity. Given the unknown distribution of the data, permutation testing was used to estimate the *P-*value (10 000 permutations), with a false discovery rate (FDR)-correction for multiple comparisons applied, *P* < 0.05.

### Machine learning analysis

A random forest (RF)- and support vector machine (SVM)-based machine learning (ML) classification modelling test was conducted to show that individuals with chronic mTBI can be reliability discriminated from those without an mTBI. Only those frequencies that showed significant between-groups effects were used. The core ML workflow followed the pipeline outlined previously by Zhang et al.^48^ Firstly, a nested 10-fold data resampling was used for feature selection (nested 10-fold FS), which utilized a recursive RF-FS (rRF-FS) algorithm.^55^ and SVM modelling. It is worth noting that the ML pipeline was modified so that no *a priori* knowledge on the ‘complete data’ was used for modelling, meaning the nested 10-fold FS was conducted on the nine data folds with ‘the full feature set’. Additionally, the final classification model was evaluated with 10-fold cross-validation (CV).

The RF-centric algorithm was chosen as it is relatively less prone to overfitting.^56^ The rRF-FS algorithm added iteration-based measures to reduce the instability due to stochastic nature of RF.^57^ SVM was used to determine the effectiveness of the feature selected by rRF-FS, as the gold standard for kernel function-based classifier.^58^ The SVM kernel functions give flexibility in processing potentially non-linearly separatable data.^58^

The overall workflow was as follows: The complete data were randomly divided into ten data folds by subjects. The nested FS process was then carried out iteratively on the nine data folds (FS training data), whose performance was in turn evaluated with the remaining one data fold (FS test data). To reduce the computational burden, an optional univariate statistics-based feature filter was used as an initial feature reduction prior to the rRF-FS and SVM selection. This initial feature reduction was used only when the univariate reduction on the complete data could separate the samples according to their groups, which was assessed by principal component analysis (PCA) clustering. In such cases, the univariate reduction was used during the nested 10-fold FS only on the FS training data. For the current study, the univariate feature reduction was carried out only for the functional connectivity data.

The nested 10-fold FS resulted in ten selected feature lists. Only the features selected at least by four and two iterations were retained as part of the consensus final feature list, for the functional connectivity and regional power data, respectively. Such a discrepancy was due to the fact that the FS process was conducted without initial univariate feature reduction for the regional power data (see above). The effectiveness of the nested 10-fold FS was assessed by sample label permutation test with SVM modelling.^59^ The modelling generalizability was determined by partial least squares-discriminant analysis (PLS-DA) and the associated sample label permutation test.^59^ A *P-*value was generated for the permutation tests also according to Ojala and Garriga.^59^ Ultimately, a final SVM classification model was generated with the consensus features. The model performances were reported as the mean CV accuracy±SD.

To compare the performance between the final regional power and functional connectivity models, we applied these models to the full subject set (i.e. the training data) as a result of the lack of naïve data. Due to the comparative nature of this analysis, the use of the training data would not impact the results. Indeed, Table 4 showed that the same results could be derived from the CV accuracies. Since the training data was used, a value close to 1 was expected for ROC-AUC (receiver operating characteristic-area under curve) analysis. For functional connectivity, the connection code and name annotation can be found in the Supplementary Material (connection_annot.csv).

### Data availability

Data are available from the corresponding author upon reasonable request.

## Results

### Mechanisms and characteristics of injury

Chronic mTBI participants reported the mechanisms of injury in the following proportions: 50% fall; 37.5% sports-related; 6.25% motor-vehicle accident; and 6.25% other. The majority of participants had at least one PPCS (87.5%), with 68.75% reporting at least two to five complaints, and 50% reporting at least six complaints. Headache and fatigue were the most common symptoms, with over 50% of participants reporting these two complaints, followed by 50% reporting irritability, difficulty concentrating 50%, feeling mentally ‘foggy’ 47%, dizziness 44%, drowsiness 40%, sensitivity to light 40%, sensitivity to noise 36%, nausea 34%, answer questions more slowly than usual 29%, difficulty remembering 27%, nervousness 27%, vision problems 27%, sadness 20%, balance problems 20%, and feeling slowed down 13%. Characteristics of injury are listed in Table 2.

### Pathological slow-wave oscillations in children and adolescents with chronic mTBI

**Figure 1:**
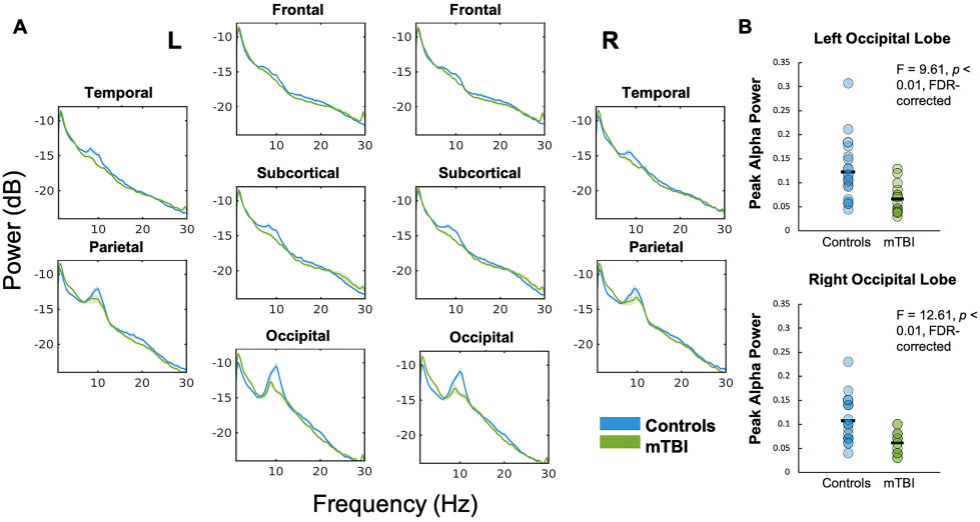
**Power spectral density plotted by hemisphere and by lobe.** The PSD is displayed for mTBI and the control group, divided by hemisphere and by lobe, for 3–30 Hz (**A**). Peak alpha power was significantly reduced in the left and right occipital lobes in the mTBI group (both *P* < 0.01, FDR-corrected) (**B**)—however, ‘broadband’ alpha was not significantly different.

**Figure 2:**
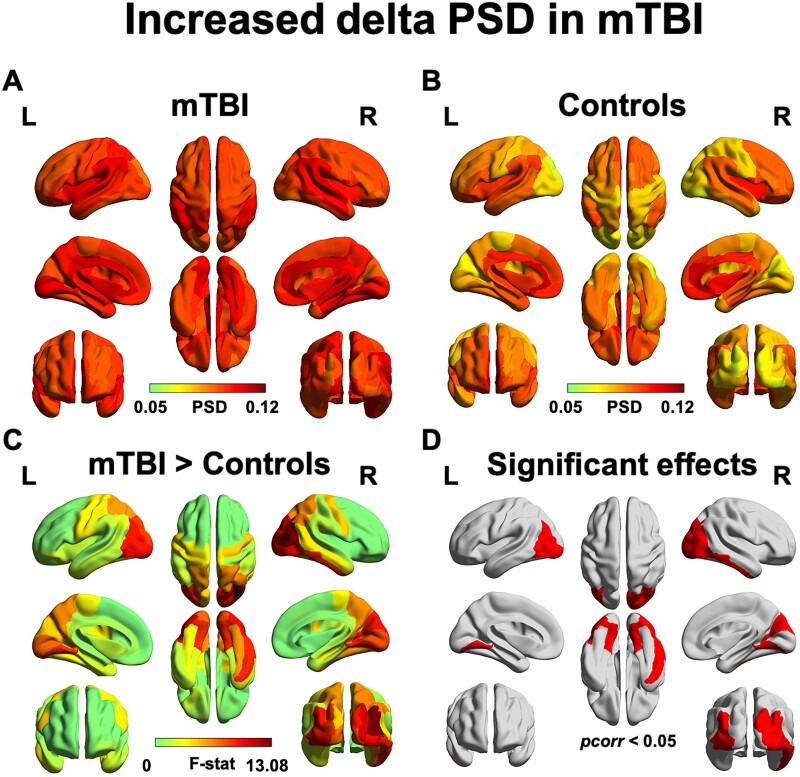
**Children and adolescents with chronic mTBI show pathological increases in delta power.** Increased power in the delta band was found in bilateral occipito-parietal areas and the right inferior temporal and somatosensory gyrus. Regional power in the mTBI (**A**) and the control groups (**B**) is displayed. The F-statistic map for group contrasts (**C**) reveal increased power in the mTBI compared to controls as confirmed by a *post hoc* Wilcoxon Rank-Sum test; binarized FDR-corrected maps showing significant regions (*pcorr* < 0.05) is plotted in red (**D**).

Assessment of the PSD, when averaged by lobe in each hemisphere, shows that ‘peak’ alpha (∼10 Hz) power was reduced in those with mTBI compared to controls, which was most pronounced in bilateral occipital lobes. Peak alpha power was significantly reduced in the left and right occipital lobes in mTBI compared to controls (both *P* < 0.01, FDR-corrected; Fig. 1). However, ‘broadband’ alpha power (8–14 Hz) was not found to be significantly reduced in mTBI. We did find that delta power was significantly increased in bilateral occipital brain regions and in the right inferior temporal gyrus compared to controls (*pcorr* < 0.05; Fig. 2). No significant differences in regional power were found in any of the other frequency bands (all *pcorr* *>* 0.05), which can be found in Supplementary Fig. 1.

### Children and adolescents with chronic mTBI show differential hypo- and hyperconnectivity

**Figure 3:**
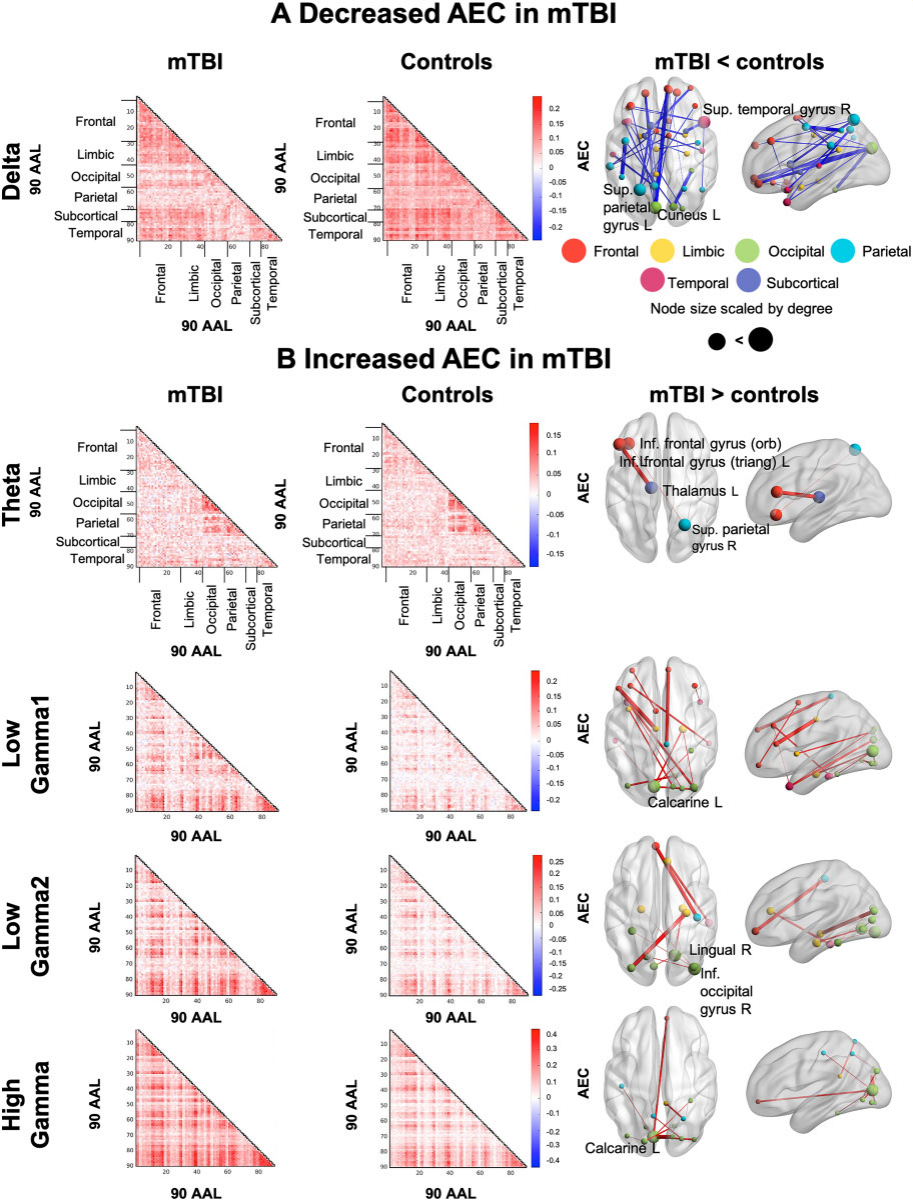
**Dysregulated functional coupling is frequency-specific in mTBI.** Significant between-group differences in functional coupling were determined by a one-way ANCOVA computed for individual AEC values, depicted as elements in the 90-by-90 connectivity matrix; permutation testing was used to estimate the *P*-value (10 000 permutations), with a FDR-correction for multiple comparisons applied across the whole connectome space (e.g. 4005 unique connections) at *P* < 0.05. Amplitude envelope correlation matrices are displayed for the chronic mTBI (left column) and control groups (second column). The glass brains (third column) show the significant differences. The width of the edges is scaled by the F-statistic, and the node size is scaled by degree. AEC is decreased in chronic mTBI vs. controls in the delta band (**A**), and increased in mTBI in theta and gamma bands (**B**).

In the delta frequency band, AEC was significantly decreased in the mTBI group compared to controls (*pcorr* < 0.05) among widespread brain areas. Reduced connectivity in mTBI primarily involved connections between bilateral prefrontal brain regions to early visual, temporal and parietal cortices, as well as between bilateral orbitofrontal brain areas to limbic and subcortical areas, including the posterior cingulate gyrus and left pallidum, respectively. Many connections also involved bilateral temporal-to-occipital and parietal brain regions. Furthermore, dysregulated connections amongst limbic areas were also observed, including the left amygdala to right hippocampus (Fig. 3A).

In the theta band, we found significantly increased coupling in those with mTBI vs. controls (*pcorr* < 0.05). Increased connectivity in the mTBI group was observed between the left triangular part of the inferior frontal gyrus and left thalamus, and the left inferior orbito-frontal gyrus and the right superior parietal gyrus (Fig. 3B).

Likewise, significantly increased AEC was observed across the gamma range, including low gamma1 (30–55 Hz), low gamma2 (65–80 Hz) and high gamma bands (80–150 Hz) in mTBI (both *pcorr* < 0.05). For low gamma1, significantly increased connectivity involved bilateral prefrontal, occipital, limbic and temporal brain areas. In particular, prefrontal brain areas were linked to right temporal areas, as well as the left middle cingulate gyrus, left supplementary motor area and right paracentral lobule. Bilateral connections within the occipital lobe were observed, as well as between occipital brain areas and left temporal and limbic regions (Fig. 3B). For low gamma2, hyperconnectivity in mTBI was observed between bilateral prefrontal areas, and right parietal and temporal regions; between bilateral occipital areas and the medial temporal lobe, including the left and right parahippocampal gyri and right hippocampus (Fig. 3B).

In high gamma, occipital, and occipito-parietal connections were dysrhythmic, anchored in the left calcarine, connecting to parietal and right superior frontal/medal orbital gyrus (Fig. 3B).

### Competitive multivariate classification: regional power or functional coupling?

A *posthoc* multivariate classification analysis was carried out to assess the most important features and their classification performance for identifying neural dysregulation in chronic mTBI. Specifically, the analysis was conducted for frequencies that significantly differed between groups, including: Delta regional power, and delta, theta, low gamma1, gamma2, and high gamma neural coupling. Based on the preliminary PCA clustering results, the analysis was conducted with initial univariate feature selection for the functional connectivity data, but without for the delta power data. Subsequently, both feature types were subject to the iterative two-step ML feature selection process. Supplementary Table 1 shows the selection counts (i.e. number of iterations selected from the second selection step) for the features selected by the first selection step, as a feature importance measure. The count numbers were used to decide the final selected feature list according to a threshold (see Materials and Methods section for details).

**Figure 4:**
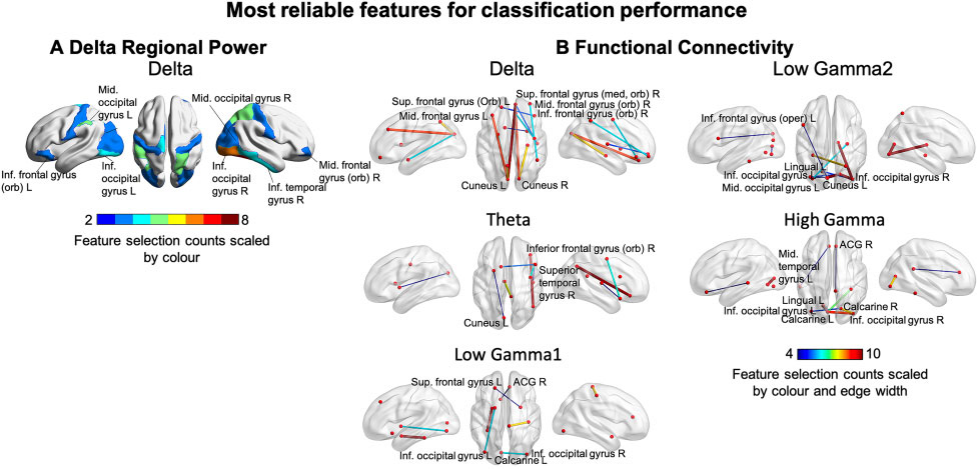
**Selected features for ML classification performance.** The most reliable selected features for ML classification performance are shown for regional power (**A**) and functional connectivity (**B**). Feature selection counts are scaled by colour and by edge width for functional connectivity and region colour for regional power.

**Figure 5:**
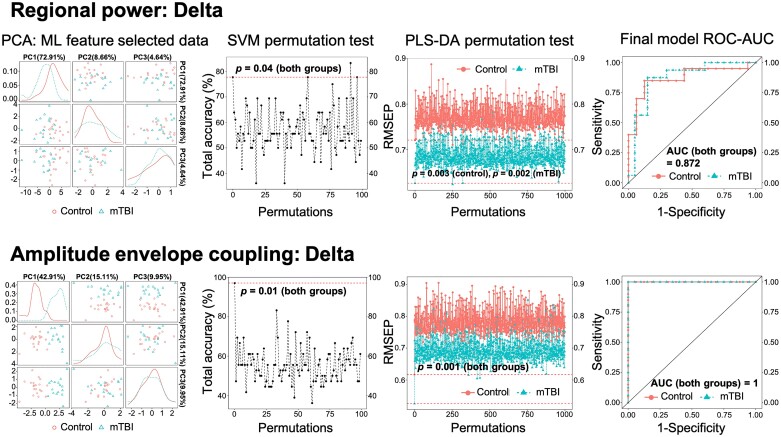
**Classification analysis shows functional connectivity outperforms regional power in classification modelling at the delta frequency.** The PCA results show that the functional connectivity data successfully separated the two participant groups with ML selected features, while the delta power data failed to achieve so. The permutation tests for both SVM and PLS-DA modelling showed that the delta functional connectivity models are more significant (smaller *P*-values) than the regional power models. When tested on the entire subject set (training data), the delta power model failed to achieve the expected optimal performance in AUC, whereas the functional connectivity counterpart achieved such.

Overall, the results exhibited ‘data type-specific’ patterns, suggesting the functional connectivity features and models substantially outperformed the regional power data for classification purposes. Specifically, the consensus functional edges selected by the nested 10-fold FS for all five frequencies led to clear group separation according to PCA clustering. Additionally, although the permutation tests suggested that selected features led to significant SVM and PLS-DA models for both data types (permutation *P-*value < 0.05), functional connectivity models exhibited higher levels of significance (i.e. smaller *P*-values) [see Fig. 4; Table 3 for the selected features for regional power (A) and functional connectivity (B)]. Relative classification performance showed that all the functional connectivity models outperformed the delta power model in terms of competitive accuracy (Table 4). Among the functional connectivity models, delta and theta coupling showed the overall best performance (97.5 ± 7.91%, mean±SD). Additionally, to compare the modelling performance between the two data types, we applied the final SVM models to the full subject data set (i.e. the training data). The ROC-AUC results suggested that the delta power model failed to achieve the perfect performance expected from the training data, while the functional connectivity model performed optimally (Fig. 5). Similar performances were observed for the functional connectivity models at the other frequency bands.

**Table 3:** List of most reliable features for machine learning classification performance

(a) Selected regions	Delta	Calcarine_L, Cingulum_Mid_L, Frontal_Inf_Orb_L, Lingual_L, Occipital_Inf_L, Occipital_Mid_L, Occipital_Sup_L, Paracentral_Lobule_L, Parietal_Inf_L, Postcentral_L, Calcarine_R, Frontal_Mid_Orb_R, Fusiform_R, Hippocampus_R, Lingual_R, Occipital_Inf_R, Occipital_Mid_R, Occipital_Sup_R, Parietal_Sup_R, Postcentral_R, Temporal_Inf_R
(b) Selected connections (node pairs)	Delta	Amygdala_L: Cuneus_L Caudate_L: Putamen_R Cuneus_L: Precuneus_L Frontal_Med_Orb_R: Heschl_R Frontal_Med_Orb_R: Cuneus_L Frontal_Mid_L: Cuneus_L Frontal_Mid_Orb_R: Parietal_Inf_R Frontal_Sup_Orb_L: Frontal_Inf_Orb_R Hippocampus_R: Cuneus_R Precentral_R: Rectus_R Rectus_R: Cuneus_R
	Theta	Cingulum_Mid_L: Cingulum_Post_R Cuneus_L: Putamen_L Frontal_Inf_Oper_R: Caudate_L Frontal_Inf_Orb_R: Angular_R Heschl_R: Temporal_Pole_Sup_R Precentral_R: Temporal_Pole_Sup_R
	Logamma1	Amygdala_L: Fusiform_L Calcarine_L: Occipital_Inf_R Frontal_Sup_L: Amygdala_R Occipital_Inf_L: Pallidum_L Olfactory_L: Cingulum_Ant_R Postcentral_R: Paracentral_Lobule_R
	Logamma2	Calcarine_L: Calcarine_R Calcarine_L: Occipital_Inf_L Calcarine_R: Occipital_Inf_L Calcarine_R: Occipital_Inf_R Frontal_Inf_Oper_L: Cuneus_L Fusiform_L: Parietal_Sup_R Hippocampus_R: Occipital_Mid_L Lingual_L: Occipital_Inf_R Occipital_Inf_R: Thalamus_R
	High gamma	Calcarine_L: Lingual_L Calcarine_L: Occipital_Inf_R Calcarine_L: Fusiform_R Calcarine_R: Occipital_Inf_L Calcarine_R: Occipital_Inf_R Cingulum_Ant_R: Cingulum_Post_R Rectus_L: Temporal_Mid_L

**Table 4:** Final model performance

Frequency	Cross-validation accuracy (%, mean±SD)	Training data accuracy (%)
Regional power		
Delta	78.3 ± 16.8	86.11
Functional connectivity		
Delta	97.5 ± 7.91	100
Theta	97.5 ± 7.91	100
Low gamma1	94.2 ± 12.5	94.4
Low gamma2	90.8 ± 13.9	94.4
High gamma	93.3 ± 14.1	100

Both cross-validation and training data accuracy are shown. The table shows low degree (functional connectivity: Delta, Theta and High gamma) of or no overfitting (Regional power, Function connectivity: Low gammas), and that functional connectivity models outperformed the delta power model.

## Discussion

The present study used non-invasive neurophysiological imaging to investigate neural functioning in children and adolescents with chronic mTBI. The majority of our chronic mTBI cases presented with PPCS, and we found pathophysiological slow-wave activity in occipito-parietal and inferior temporal areas, as well as dysrhythmic ‘peak’ alpha power in occipital regions, with significant reductions in mTBI—a phenomenon previously linked to de-afferentation, demyelination and axonal injury, as well as pointing to potential dysfunction in thalamic pacemaker cells and disinhibited visual cortices. Furthermore, we found patterns of frequency-specific increases and decreases in functional coupling in distributed neural circuits. Comparatively, functional coupling indices provided comparatively more reliable features for classification modelling. Chronic mTBI accompanied by PPCS can be debilitating and severely impact an individual’s quality of life. It is particularly unfortunate for children and adolescents, during a time of rapid neurodevelopmental and psychosocial change—these ‘invisible oscillopathies’ increasingly appear to be reliable biomarkers of a dysregulated neural repertoire in ‘mild’ brain injury, and potentially offer a route to precision clinical interventions and predictors of treatment response.

### Pathological slow-wave oscillations and dysregulated alpha activity in children and adolescents with chronic mTBI

Our findings of elevated delta power—a ‘slowing’ in rhythmic neural activity—in children and adolescents with chronic mTBI are consistent with previous reports of potentiated low-frequency activity in adults with concussion.^21^^,^^23–27^ Abnormal activity in the delta range has been shown to be generated by cortical grey matter areas that have endured de-afferentation and demyelination as a result of damage to white matter fibres, caused by diffuse and focal axonal injury.^10^^,^^11^^,^^23^^,^^60^ In addition to axonal injury, abnormal generation of delta in mTBI might be driven by interference to cholinergic transmission following injury.^61^ This hypothesis is supported by animal research demonstrating that irregular delta oscillations, like those generated by axonal injury, can be induced via atropine administration disrupting cholinergic transmission.^62^

More recently, evidence suggests that the pathological slowing of rhythmic brain activity in mTBI may actually be indicative of neuronal healing and repair processes.^63^ Delta oscillations prevalent during sleep and anaesthesia are thought to reflect the clearance of beta-amyloid and beta-amyloid precursor proteins, which are established markers of axonal injury in mTBI.^63–67^ In rodents with mTBI, sleep-induced delta activity reduced accumulation of beta-amyloid precursor protein and prevented cognitive deficit following injury, suggesting a potential roleof slowing of neural dynamics as a marker of neuroprotection.^63^ Thus, in cases of chronic mTBI ‘pathological’ delta may be a marker of clearance mechanisms of neural toxins and repair and restoration after injury.

Additionally, ‘peak’ alpha activity was suppressed in bilateral occipital cortices in chronic mTBI. Attenuated occipital alpha has previously been shown to be diminished in adults with mTBI in the early stages of injury.^68^ Like pathological slow-wave activity in mTBI, reduced alpha peak power is linked to damage to white matter tracts, consistent with axonal injury,^69^^,^^70^ and points to disrupted thalamic pace maker cell functioning and/or reciprocal thalamo-cortical connectivity.^71^ Additionally, alpha suppression points to disinhibited and/or hyperexcitability as a consequence of injury, converging with cross-modal evidence of damage-dependent changes in cortical inhibition and excitability in youth with chronic mTBI.^72^

### Dysregulated large-scale circuits and connectivity in chronic mTBI

We are the first to demonstrate disrupted neurophysiological coupling and communication in children and adolescents with chronic mTBI. Like adults with injury, frequency-specific patterns of both increased and decreased functional coupling were observed, with ‘reduced’ delta connectivity, the direction of effect in opposition to the regional power results. Conversely, we observed increased functional coupling in the theta and gamma bands in mTBI. These findings suggest mTBI differentially and insidiously impacts multiplexing, frequency-dependent channels of neural communication and coherence in the brain, and has a pernicious and lasting effect on the brain health in children and adolescents; these effects are not restricted to focal and localized disruption, but impact the distributed coordination of regional neural dynamics, over a year, on average, after injury.

### Mechanisms of altered connectivity in mTBI

Reduced functional connectivity was observed in the delta frequency range in the mTBI group. Diffuse micro-lesions in white matter tracts (i.e. diffuse axonal injury) resulting from biomechanical forces of injury are known to disrupt both structural and functional large-scale networks.^73–75^ We have previously suggested that dysregulated neural connectivity in adults with mTBI may reflect micro-alterations in white matter, that in turn, may lead to persistent cognitive, physical, behavioural, and emotional symptoms.^21^^,^^76^ Therefore, we speculate that our findings of neural dysconnectivity, in the delta range, may be linked to widespread alterations in the microstructure of white matter pathways.

Additionally, we found elevated functional coupling in the theta and gamma ranges in children and adolescents with mTBI. One possible explanation for increased widespread connectivity, particularly in the gamma range, may be due to an imbalance in the ratio of excitatory/inhibitory (E/I) drive as a result of neuronal damage (see Ref. 77) for a review. This shift reflects a reduction in GABAergic interneurons or damage to their receptors, resulting in GABAergic dysregulation and disinhibition and potentiated glutamatergic excitation.^77–79^ This altered e:i neurotransmitter balance has been suggested in transcranial magnetic stimulation studies of children with mTBI.^72^^,^^80^ Since the generation and temporal dynamics of coordinated gamma oscillations rely on parvalbumin-positive GABA interneuron inhibition,^81^^,^^82^ neuronal cell death or damage could lead to maladaptive local and long-range gamma functional coupling and dynamics.^77^^,^^83^ Huang and colleagues^30^ have proposed that reduced inhibition/increased excitation may drive increased gamma coupling in adults with mTBI—thus, it seems reasonable that a similar putative mechanism is at play in the observation made here.

Amplitude envelope coupling reliably recapitulates the intrinsic functional architecture defined by slower haemodynamic activity (an indirect correlate of electrophysiological activity) in functional magnetic imaging (fMRI) blood-oxygenation-level-dependent (BOLD) networks.^34^^,^^54^^,^^84^ A large body of resting-state fMRI studies have reported abnormal patterns of both reduced and increased functional connectivity within and between intrinsic networks in individuals with PPCS following brain injury, showing altered connectivity is related to cognitive and affective sequalae.^85^^,^^86^ Our findings of dysrhythmic neural coupling in chronic mTBI complement and support prior haemodynamic studies.

### Frontal, temporal and occipital disrupted coupling

Abnormal hyper- and hypo-functional coupling in mTBI was primarily found in frontal, temporal and occipital brain areas. Altered connectivity of these brain areas is consistent with previous reports in adults with mTBI,^21^^,^^32^ and the idea that these areas may be particularly susceptible to injury.^61^ In addition, we found altered connectivity involving limbic brain areas, including the hippocampus, parahippocampus and amygdala. Cortico-limbic circuitry is critically involved in emotion^87^ and memory processing,^88^ and dysconnectivity among limbic areas has been observed in adults with mTBI.^30^

### Competitive classification: regional power or coupling?

By integrating a multivariate classification analysis with neurophysiological imaging, we can accurately identify individual youth with chronic mTBI. Comparatively speaking, classification accuracy was higher when using functional connectome data compared to delta regional power. This finding is supported by recent work from our group showing that combined functional connectome and SVM-based multivariate classification analysis provides greater discriminative power in classifying adults with mTBI from typical controls, versus regional power.^36^ Similar to adults with injury, paediatric mTBI consequently results in the dysregulation of rhythmic neural firing, in turn disrupting the dynamic segregation and integration of distributed brain regions. This is consistent with the notion that large-scale neural networks are especially susceptible to mTBI, and are not just a discrete focal injury (e.g. coup-contrecoup). Of course, our limited sample size brings with it the caveat that some of the models exhibited a low degree of overfitting, but encouragingly, these results suggest that also framing mTBI as a network disorder, rather than just a focal injury, would to some degree explain the diverse symptom profiles observed in chronic mTBI.

A reliable and objective instrument for understanding the pathophysiology of mTBI is needed.^89^^,^^90^ The clinical symptoms of mTBI are broad and heterogenous, with substantial inter-individual variability and diversity, a combination of the type of injury and pre-existing circumstances contributing to profiles and eventual outcome. Varied reports of dysregulated brain activity, and discrepancies in the relationship between mTBI, PPCS and neural dysregulation following injury are testament to the substantial heterogeneity of symptoms and outcomes.^91^ Using methodologies that capture and integrate this broad heterogeneity will be the only way we can effectively diagnose and treat individuals with mTBI and PPCS.^91^ Our classification technique integrated with high-resolution neurophysiological imaging offers a promising approach to accurately and objectively classifying cases of ‘invisible’ and ‘mild’ brain injury in children and adolescents.

### Future directions and conclusions

For the first time, we show that there is a simultaneous slowing of rhythmic neural activity and disrupted functional coupling in children and adolescents after mTBI, on average a year after injury, and that the majority report persistent symptoms. We also demonstrate that the integration of our classification approach with functional connectome data can accurately discriminate those with injury from typically developing controls, with a better classification performance than regional power alone. In the present study, the majority, but not all participants report enduring symptoms; we acknowledge that participant recruitment may have been biased by participant self-selection and were predominantly symptomatic when volunteering. Thus, an important future direction will be to study differences in local and long-range functional coupling in three groups: Children and adolescents with mTBI with PPCS, those without PPCS, and typically developing controls. An objective test of whether distinct neurophysiological profiles can discriminate these groups will be to apply our classification approach. A potential limitation of the present study is the confound of head movement—this nuisance covariate was not accounted for when calculating the AEC. Accouting for it may attenuate artefactual ‘ghost’ interactions, with motion otherwise spuriously inflatingd connectivity estimates^92^; it will be an important step to implement this in future studies. Furthermore, future studies should examine acute and subacute phases of injury, as the trajectories of recovery and brain activity are highly dynamic.^93^ Moreover, the dynamic repertoire of brain activity is known to be a strong predictor of mTBI recovery and would be an important biomarker to understand the pathophysiology of injury.

## Conclusion

Our results show that localized and interregional neural function is altered in children and adolescents with chronic mTBI, the majority presenting enduring symptoms. Given the adverse developmental consequences of chronic mTBI and associated PPCS in youth, a clearer understanding of the lingering neurophysiological disruption after injury is crucial for predicting who will go on to develop persistent symptoms following injury, as well as implementing targeted-treatment interventions in pursuit of precision medicine. Furthermore, we show that the functional connectome is a robust and accurate classification marker for paediatric mild traumatic brain injury, and that these ‘invisible oscillopathies’ reflect persistent neural network dysfunction and a potential hallmark of the disorder.

## Supplementary material

Supplementary material is available at *Brain Communications* online.

## Funding

This study was funded by the Hospital for Sick Children Foundation and Centre for Brain and Mental Health ‘Chase an Idea’ Grant, Brain Canada and Restracomp funding award.

## Competing interests

The authors report no competing interests.

## Supplementary Material

fcab044_Supplementary_Data

## Abbreviations

AAL =: automated anatomical labelling
AEC =: amplitude envelope correlations
ANCOVA =: analysis of covariance
BOLD =: blood-oxygenation-level-dependent
CT =: computed tomography
EEG =: electroencephalography
E/I =: excitatory/inhibitory
FDR =: false discovery rate
fMRI =: functional magnetic resonance imaging
GABA =: gamma aminobutyric acid
LCMV =: linearly constrained minimum variance
MEG =: magnetoencephalography
ML =: machine learning
MRI =: magnetic resonance imaging
mTBI =: mild traumatic brain injury
PLS-DA =: partial least squares-discriminant analysis
PPCS =: persistent post-concussive symptoms
PCSI =: Post-Concussion Symptom Inventory
PSD =: power spectral density
PCA =: principal component analysis
RF =: random forest
ROC-AUC =: receiver operating characteristic-area under curve
rRF-FS =: recursive RF-FS
SQUID =: superconducting quantum interference device
SVM =: support vector machine
