# Supplementary material for: Mild traumatic brain injury is associated with dysregulated neural network functioning in children and adolescents
Source: Brain Commun. 2021 Mar 17;3(2):fcab044. doi: 10.1093/braincomms/fcab044 (PMC8176148; doi:10.1093/braincomms/fcab044)
Supplement: fcab044_Supplementary_Data [file fcab044_supplementary_data.zip › Supplementary_Material.docx]

**Supplemental Materials**

**
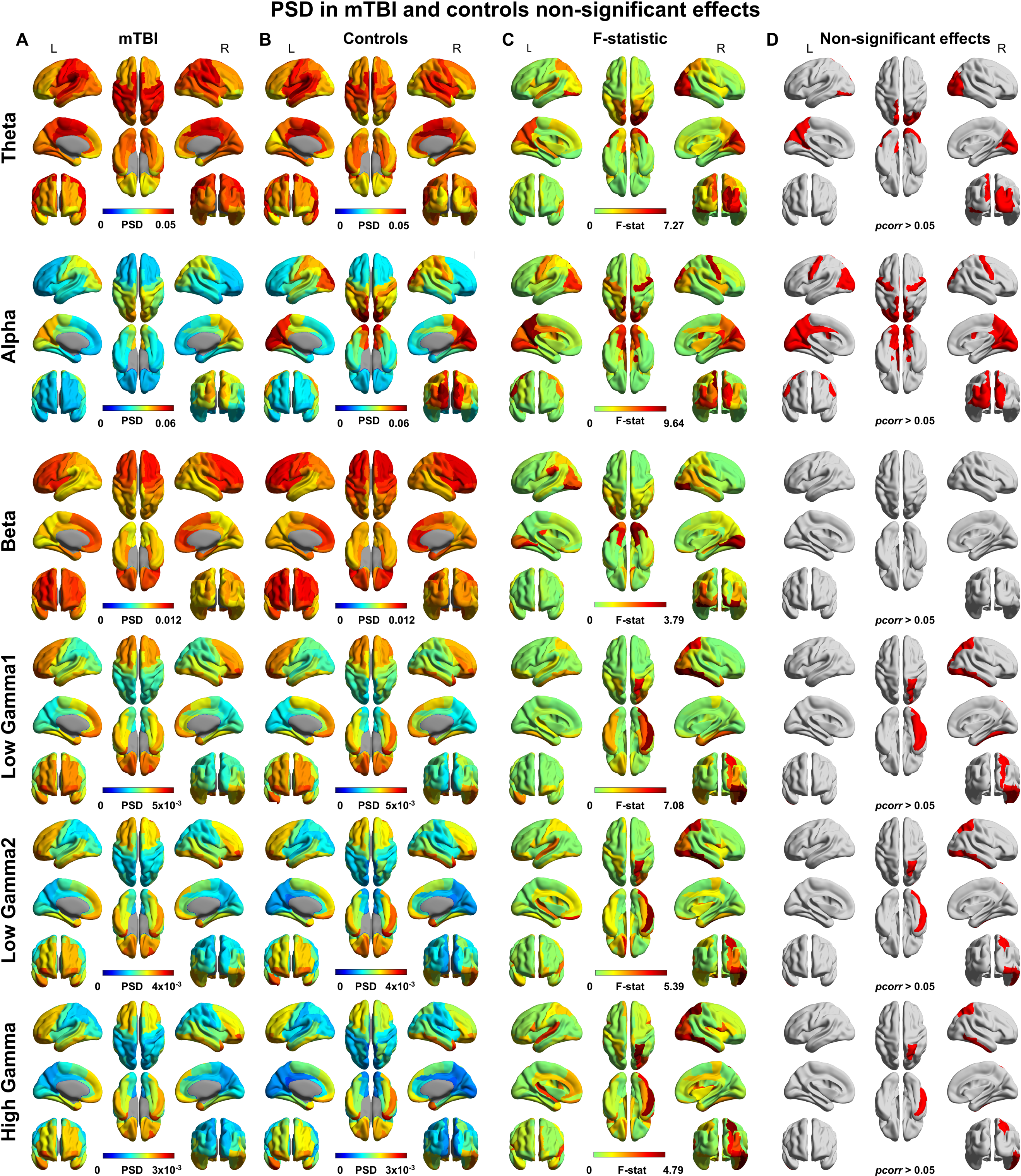
**

**Supplemental Figure 1. Power spectral density in children and adolescents with mTBI and controls.** Regional power in the mTBI (A) and the control groups (B) is shown. The F-statistic map for group contrasts (C) reveal non-significant between-group effects for the theta, alpha, beta and gamma bands; binarized FDR-corrected maps showing non-significant regions (all *pcorr* > 0.05) is plotted in red (D).

**Supplemental Table 1. Selected Feature Counts**

|  | Features | Count |
| --- | --- | --- |
| Delta; | Frontal_Med_Orb_R: Cuneus_L | 10 |
| Selected | Rectus_R: Cuneus_R | 9 |
| connections | Frontal_Mid_L: Cuneus_L | 9 |
| (node pairs) | Hippocampus_R: Cuneus_R | 8 |
|  | Cuneus_L: Precuneus_L | 8 |
|  | Frontal_Mid_Orb_R: Parietal_Inf_R | 6 |
|  | Precentral_R: Rectus_R | 6 |
|  | Amygdala_L: Cuneus_L | 6 |
|  | Frontal_Med_Orb_R: Heschl_R | 5 |
|  | Frontal_Sup_Orb_L: Frontal_Inf_Orb_R | 5 |
|  | Caudate_L: Putamen_R | 4 |
|  | Precentral_L: Cuneus_R | 3 |
|  | Frontal_Mid_Orb_R: Occipital_Sup_R | 3 |
|  | Frontal_Mid_Orb_R: Occipital_Mid_L | 3 |
|  | Frontal_Sup_Medial_L: Thalamus_R | 3 |
|  | Calcarine_L: Cuneus_L | 3 |
|  | Cuneus_L: Caudate_R | 3 |
|  | Precentral_L: ParaHippocampal_L | 2 |
|  | Frontal_Mid_Orb_R: Cuneus_L | 2 |
|  | Frontal_Inf_Oper_L: Cingulum_Post_L | 2 |
|  | Insula_L: Caudate_R | 2 |
|  | ParaHippocampal_L: Temporal_Sup_R | 2 |
|  | Amygdala_L: Occipital_Inf_R | 2 |
|  | Cuneus_R: Fusiform_R | 2 |
|  | Cuneus_R: Temporal_Pole_Mid_L | 2 |
|  | Putamen_R: Pallidum_L | 2 |
|  | Precentral_L: Putamen_L | 1 |
|  | Frontal_Mid_Orb_R: Parietal_Sup_R | 1 |
|  | Frontal_Inf_Oper_L: Occipital_Sup_L | 1 |
|  | Frontal_Inf_Tri_R: Caudate_R | 1 |
|  | Frontal_Sup_Medial_L: Occipital_Mid_L | 1 |
|  | Frontal_Sup_Medial_R: Temporal_Pole_Sup_L | 1 |
|  | Frontal_Med_Orb_R: Parietal_Sup_L | 1 |
|  | Cingulum_Ant_R: Fusiform_L | 1 |
|  | Hippocampus_R: Caudate_R | 1 |
|  | Frontal_Sup_R: Cuneus_L | 1 |
|  | Cuneus_L: Putamen_L | 1 |
|  | Frontal_Sup_Orb_L: Cuneus_L | 1 |
|  | Frontal_Sup_Orb_L: Angular_L | 1 |
|  | Frontal_Sup_Orb_L: Paracentral_Lobule_R | 1 |
|  | Occipital_Sup_R: Caudate_R | 1 |
|  | Occipital_Mid_R: Temporal_Sup_R | 1 |
|  | Parietal_Sup_L: Caudate_R | 1 |
|  | Parietal_Inf_L: SupraMarginal_R | 1 |
|  | Heschl_R: Temporal_Pole_Sup_L | 1 |
| Theta | Frontal_Inf_Orb_R: Angular_R | 10 |
|  | Cingulum_Mid_L: Cingulum_Post_R | 8 |
|  | Precentral_R: Temporal_Pole_Sup_R | 7 |
|  | Frontal_Inf_Oper_R: Caudate_L | 6 |
|  | Cuneus_L: Putamen_L | 5 |
|  | Heschl_R: Temporal_Pole_Sup_R | 5 |
|  | Insula_R: Calcarine_R | 3 |
|  | Rolandic_Oper_L: Amygdala_R | 2 |
|  | ParaHippocampal_R: Amygdala_L | 2 |
|  | Paracentral_Lobule_L: Pallidum_L | 2 |
|  | Frontal_Mid_L: Occipital_Mid_L | 2 |
|  | Thalamus_R: Temporal_Inf_R | 2 |
|  | Frontal_Mid_Orb_R: Lingual_R | 1 |
|  | Frontal_Inf_Oper_L: SupraMarginal_R | 1 |
|  | Frontal_Inf_Oper_L: Thalamus_L | 1 |
|  | Frontal_Inf_Tri_R: Olfactory_R | 1 |
|  | Frontal_Inf_Tri_R: Putamen_L | 1 |
|  | Frontal_Inf_Orb_L: Postcentral_L | 1 |
|  | Rolandic_Oper_L: Postcentral_R | 1 |
|  | Supp_Motor_Area_L: Putamen_R | 1 |
|  | Supp_Motor_Area_R: Amygdala_L | 1 |
|  | Supp_Motor_Area_R: Occipital_Sup_R | 1 |
|  | Supp_Motor_Area_R: Occipital_Mid_L | 1 |
|  | Olfactory_L: Temporal_Pole_Sup_R | 1 |
|  | Olfactory_R: Thalamus_R | 1 |
|  | Rectus_R: Temporal_Sup_L | 1 |
|  | Cingulum_Ant_R: Precuneus_R | 1 |
|  | Cingulum_Ant_R: Temporal_Pole_Sup_R | 1 |
|  | Cingulum_Mid_L: Temporal_Pole_Mid_L | 1 |
|  | Cingulum_Mid_R: Occipital_Mid_R | 1 |
|  | Hippocampus_L: Pallidum_R | 1 |
|  | Hippocampus_L: Temporal_Pole_Mid_R | 1 |
|  | Amygdala_L: Temporal_Mid_L | 1 |
|  | Lingual_L: Precuneus_R | 1 |
|  | Lingual_L: Temporal_Mid_R | 1 |
|  | Lingual_L: Temporal_Inf_R | 1 |
|  | Lingual_R: Paracentral_Lobule_R | 1 |
|  | Fusiform_L: Thalamus_R | 1 |
|  | Fusiform_R: Temporal_Mid_L | 1 |
|  | Frontal_Sup_Orb_R: Rolandic_Oper_L | 1 |
|  | Parietal_Sup_R: Pallidum_R | 1 |
|  | Parietal_Inf_R: Heschl_L | 1 |
|  | SupraMarginal_R: Heschl_R | 1 |
|  | Precuneus_L: Temporal_Pole_Mid_L | 1 |
|  | Precuneus_R: Paracentral_Lobule_L | 1 |
|  | Frontal_Mid_L: ParaHippocampal_L | 1 |
|  | Frontal_Mid_L: Temporal_Mid_L | 1 |
|  | Paracentral_Lobule_R: Pallidum_R | 1 |
|  | Thalamus_R: Temporal_Pole_Mid_L | 1 |
|  | Heschl_L: Temporal_Pole_Mid_R | 1 |
|  | Frontal_Mid_R: Frontal_Sup_Medial_R | 1 |
|  | Frontal_Mid_Orb_L: Frontal_Inf_Oper_R | 1 |
|  | Frontal_Mid_Orb_L: Supp_Motor_Area_L | 1 |
| Low Gamma1 | Amygdala_L: Fusiform_L | 7 |
|  | Postcentral_R: Paracentral_Lobule_R | 6 |
|  | Calcarine_L: Occipital_Inf_R | 5 |
|  | Occipital_Inf_L: Pallidum_L | 5 |
|  | Olfactory_L: Cingulum_Ant_R | 4 |
|  | Frontal_Sup_L: Amygdala_R | 4 |
|  | Frontal_Inf_Orb_R: Caudate_L | 3 |
|  | Cingulum_Ant_L: Hippocampus_L | 3 |
|  | Lingual_R: Occipital_Inf_L | 3 |
|  | Parietal_Inf_R: SupraMarginal_R | 3 |
|  | Frontal_Mid_R: Temporal_Pole_Mid_R | 3 |
|  | Frontal_Mid_R: Frontal_Mid_Orb_L | 3 |
|  | Amygdala_L: Calcarine_R | 2 |
|  | Calcarine_R: Temporal_Inf_L | 2 |
|  | Occipital_Inf_L: Temporal_Pole_Sup_L | 2 |
|  | Occipital_Inf_R: Parietal_Inf_L | 2 |
|  | Fusiform_L: Precuneus_L | 2 |
|  | Precuneus_R: Temporal_Pole_Sup_R | 2 |
|  | Precentral_L: Postcentral_R | 1 |
|  | Frontal_Inf_Orb_L: Temporal_Mid_R | 1 |
|  | Supp_Motor_Area_L: Parietal_Inf_L | 1 |
|  | Frontal_Sup_Medial_R: Paracentral_Lobule_R | 1 |
|  | Frontal_Med_Orb_R: SupraMarginal_R | 1 |
|  | Insula_R: Hippocampus_L | 1 |
|  | Cingulum_Ant_L: Hippocampus_R | 1 |
|  | Cingulum_Ant_R: Cingulum_Mid_L | 1 |
|  | Cingulum_Mid_L: Parietal_Inf_R | 1 |
|  | Hippocampus_L: Occipital_Sup_R | 1 |
|  | Amygdala_L: Temporal_Sup_L | 1 |
|  | Calcarine_L: Occipital_Inf_L | 1 |
|  | Calcarine_R: Occipital_Inf_R | 1 |
|  | Frontal_Sup_Orb_L: Rolandic_Oper_L | 1 |
|  | Occipital_Sup_R: Caudate_R | 1 |
|  | Occipital_Sup_R: Temporal_Pole_Mid_L | 1 |
|  | Occipital_Mid_R: Occipital_Inf_R | 1 |
|  | Postcentral_L: Parietal_Inf_R | 1 |
|  | Frontal_Sup_Orb_R: Rectus_L | 1 |
|  | Angular_R: Paracentral_Lobule_L | 1 |
|  | Precuneus_R: Temporal_Sup_R | 1 |
|  | Heschl_R: Temporal_Sup_R | 1 |
|  | Temporal_Sup_R: Temporal_Mid_L | 1 |
| Low Gamma2 | Calcarine_L: Calcarine_R | 10 |
|  | Calcarine_R: Occipital_Inf_R | 10 |
|  | Occipital_Inf_R: Thalamus_R | 10 |
|  | Fusiform_L: Parietal_Sup_R | 8 |
|  | Hippocampus_R: Occipital_Mid_L | 6 |
|  | Calcarine_R: Occipital_Inf_L | 5 |
|  | Lingual_L: Occipital_Inf_R | 5 |
|  | Frontal_Inf_Oper_L: Cuneus_L | 4 |
|  | Calcarine_L: Occipital_Inf_L | 4 |
|  | ParaHippocampal_L: Occipital_Inf_L | 3 |
|  | Lingual_L: Parietal_Sup_L | 3 |
|  | Parietal_Sup_L: Parietal_Inf_L | 3 |
|  | Frontal_Inf_Tri_L: Caudate_L | 2 |
|  | Rolandic_Oper_L: ParaHippocampal_R | 2 |
|  | Frontal_Sup_Medial_L: Cingulum_Post_L | 2 |
|  | Frontal_Med_Orb_L: Postcentral_R | 2 |
|  | ParaHippocampal_L: Occipital_Inf_R | 2 |
|  | Calcarine_L: Fusiform_R | 2 |
|  | Calcarine_R: Fusiform_L | 2 |
|  | Occipital_Inf_L: Occipital_Inf_R | 2 |
|  | Frontal_Mid_Orb_L: Frontal_Inf_Oper_R | 2 |
|  | Precentral_L: Precuneus_R | 1 |
|  | Frontal_Inf_Oper_L: Occipital_Sup_L | 1 |
|  | Frontal_Inf_Tri_L: Hippocampus_L | 1 |
|  | Supp_Motor_Area_L: Fusiform_L | 1 |
|  | Precentral_R: Cingulum_Post_L | 1 |
|  | Olfactory_R: Thalamus_R | 1 |
|  | Frontal_Sup_Medial_L: Thalamus_R | 1 |
|  | Frontal_Med_Orb_R: Fusiform_L | 1 |
|  | Frontal_Med_Orb_R: Temporal_Pole_Sup_L | 1 |
|  | Rectus_R: Putamen_R | 1 |
|  | Insula_L: Temporal_Inf_L | 1 |
|  | Insula_R: Caudate_R | 1 |
|  | Cingulum_Ant_L: Cingulum_Mid_L | 1 |
|  | Cingulum_Ant_R: Cingulum_Post_R | 1 |
|  | ParaHippocampal_L: Cuneus_L | 1 |
|  | Frontal_Sup_R: Frontal_Sup_Orb_R | 1 |
|  | Cuneus_L: Occipital_Mid_R | 1 |
|  | Lingual_L: Lingual_R | 1 |
|  | Lingual_R: Occipital_Mid_R | 1 |
|  | Lingual_R: Putamen_R | 1 |
|  | Occipital_Sup_R: Caudate_L | 1 |
|  | Occipital_Mid_R: Fusiform_L | 1 |
|  | Occipital_Inf_L: Temporal_Pole_Mid_R | 1 |
|  | Frontal_Sup_Orb_R: Lingual_L | 1 |
|  | Frontal_Sup_Orb_R: Pallidum_L | 1 |
|  | Parietal_Sup_R: SupraMarginal_R | 1 |
|  | Parietal_Sup_R: Temporal_Pole_Sup_L | 1 |
|  | Putamen_R: Temporal_Sup_L | 1 |
|  | Frontal_Mid_R: Olfactory_R | 1 |
|  | Frontal_Mid_Orb_L: Cingulum_Mid_L | 1 |
| High Gamma | Calcarine_L: Lingual_L | 10 |
|  | Calcarine_L: Occipital_Inf_R | 9 |
|  | Calcarine_R: Occipital_Inf_R | 8 |
|  | Calcarine_L: Fusiform_R | 7 |
|  | Calcarine_R: Occipital_Inf_L | 5 |
|  | Rectus_L: Temporal_Mid_L | 4 |
|  | Cingulum_Ant_R: Cingulum_Post_R | 4 |
|  | Frontal_Med_Orb_R: Rectus_L | 3 |
|  | Calcarine_L: Parietal_Inf_R | 3 |
|  | Rectus_L: Insula_L | 2 |
|  | Calcarine_L: Occipital_Inf_L | 2 |
|  | Lingual_L: Occipital_Sup_L | 2 |
|  | Precuneus_L: Thalamus_R | 2 |
|  | Frontal_Inf_Oper_L: Postcentral_L | 1 |
|  | Frontal_Inf_Tri_L: Pallidum_L | 1 |
|  | Frontal_Inf_Orb_R: Rolandic_Oper_L | 1 |
|  | Frontal_Inf_Orb_R: Hippocampus_L | 1 |
|  | Rolandic_Oper_R: Postcentral_L | 1 |
|  | Rolandic_Oper_R: Temporal_Sup_L | 1 |
|  | Precentral_R: Lingual_L | 1 |
|  | Olfactory_R: Rectus_L | 1 |
|  | Rectus_L: Hippocampus_L | 1 |
|  | Rectus_L: SupraMarginal_L | 1 |
|  | Rectus_L: Temporal_Pole_Mid_L | 1 |
|  | Insula_L: Amygdala_L | 1 |
|  | Cingulum_Mid_L: Cingulum_Post_L | 1 |
|  | Cingulum_Post_L: Hippocampus_L | 1 |
|  | Hippocampus_L: Putamen_L | 1 |
|  | Hippocampus_L: Pallidum_L | 1 |
|  | ParaHippocampal_L: Parietal_Inf_R | 1 |
|  | Frontal_Sup_R: Frontal_Mid_R | 1 |
|  | Calcarine_L: Temporal_Inf_R | 1 |
|  | Calcarine_R: Lingual_R | 1 |
|  | Calcarine_R: Occipital_Mid_L | 1 |
|  | Calcarine_R: Paracentral_Lobule_L | 1 |
|  | Cuneus_R: Fusiform_L | 1 |
|  | Lingual_L: Occipital_Inf_L | 1 |
|  | Occipital_Mid_L: Occipital_Inf_L | 1 |
|  | Occipital_Mid_R: Angular_L | 1 |
|  | Postcentral_L: Temporal_Sup_R | 1 |
|  | Postcentral_R: Parietal_Inf_L | 1 |
|  | Parietal_Inf_L: Temporal_Mid_R | 1 |
|  | Parietal_Inf_R: Paracentral_Lobule_R | 1 |
|  | Paracentral_Lobule_L: Temporal_Sup_L | 1 |
|  | Putamen_L: Temporal_Pole_Mid_L | 1 |
|  | Frontal_Mid_Orb_L: Frontal_Med_Orb_R | 1 |
| PSD Delta; | Lingual_L | 8 |
| Selected | Fusiform_R | 7 |
| regions | Occipital_Inf_R | 6 |
|  | Calcarine_R | 4 |
|  | Parietal_Inf_L | 4 |
|  | Parietal_Sup_R | 4 |
|  | Occipital_Inf_L | 3 |
|  | Paracentral_Lobule_L | 3 |
|  | Temporal_Inf_R | 3 |
|  | Calcarine_L | 2 |
|  | Cingulum_Mid_L | 2 |
|  | Frontal_Inf_Orb_L | 2 |
|  | Frontal_Mid_Orb_R | 2 |
|  | Hippocampus_R | 2 |
|  | Lingual_R | 2 |
|  | Occipital_Mid_L | 2 |
|  | Occipital_Mid_R | 2 |
|  | Occipital_Sup_L | 2 |
|  | Occipital_Sup_R | 2 |
|  | Postcentral_L | 2 |
|  | Postcentral_R | 2 |
|  | Amygdala_L | 1 |
|  | Amygdala_R | 1 |
|  | Angular_L | 1 |
|  | Angular_R | 1 |
|  | Caudate_L | 1 |
|  | Caudate_R | 1 |
|  | Cingulum_Ant_L | 1 |
|  | Cingulum_Ant_R | 1 |
|  | Cingulum_Mid_R | 1 |
|  | Cingulum_Post_L | 1 |
|  | Cingulum_Post_R | 1 |
|  | Cuneus_L | 1 |
|  | Cuneus_R | 1 |
|  | Frontal_Inf_Oper_L | 1 |
|  | Frontal_Inf_Oper_R | 1 |
|  | Frontal_Inf_Orb_R | 1 |
|  | Frontal_Inf_Tri_L | 1 |
|  | Frontal_Inf_Tri_R | 1 |
|  | Frontal_Med_Orb_L | 1 |
|  | Frontal_Med_Orb_R | 1 |
|  | Frontal_Mid_L | 1 |
|  | Frontal_Mid_Orb_L | 1 |
|  | Frontal_Mid_R | 1 |
|  | Frontal_Sup_L | 1 |
|  | Frontal_Sup_Medial_L | 1 |
|  | Frontal_Sup_Medial_R | 1 |
|  | Frontal_Sup_Orb_L | 1 |
|  | Frontal_Sup_Orb_R | 1 |
|  | Frontal_Sup_R | 1 |
|  | Fusiform_L | 1 |
|  | Heschl_L | 1 |
|  | Heschl_R | 1 |
|  | Hippocampus_L | 1 |
|  | Insula_L | 1 |
|  | Insula_R | 1 |
|  | Olfactory_L | 1 |
|  | Olfactory_R | 1 |
|  | Pallidum_L | 1 |
|  | Pallidum_R | 1 |
|  | Paracentral_Lobule_R | 1 |
|  | ParaHippocampal_L | 1 |
|  | ParaHippocampal_R | 1 |
|  | Parietal_Inf_R | 1 |
|  | Parietal_Sup_L | 1 |
|  | Precentral_L | 1 |
|  | Precentral_R | 1 |
|  | Precuneus_L | 1 |
|  | Precuneus_R | 1 |
|  | Putamen_L | 1 |
|  | Putamen_R | 1 |
|  | Rectus_L | 1 |
|  | Rectus_R | 1 |
|  | Rolandic_Oper_L | 1 |
|  | Rolandic_Oper_R | 1 |
|  | Supp_Motor_Area_L | 1 |
|  | Supp_Motor_Area_R | 1 |
|  | SupraMarginal_L | 1 |
|  | SupraMarginal_R | 1 |
|  | Temporal_Inf_L | 1 |
|  | Temporal_Mid_L | 1 |
|  | Temporal_Mid_R | 1 |
|  | Temporal_Pole_Mid_L | 1 |
|  | Temporal_Pole_Mid_R | 1 |
|  | Temporal_Pole_Sup_L | 1 |
|  | Temporal_Pole_Sup_R | 1 |
|  | Temporal_Sup_L | 1 |
|  | Temporal_Sup_R | 1 |
|  | Thalamus_L | 1 |
|  | Thalamus_R | 1 |
